# Supplementary material for: Aminobisphosphonates reactivate the latent reservoir in people living with HIV-1
Source: bioRxiv. 2023 Feb 7:2023.02.07.527421. Preprint. [Version 1] doi: 10.1101/2023.02.07.527421 (PMC9934553; doi:10.1101/2023.02.07.527421)
Supplement: Supplement 1 [file NIHPP2023.02.07.527421v1-supplement-1.pdf]

bioRxiv preprint doi: https://doi.org/10.1101/2023.02.07.527421; this version posted February 7, 2023. The copyright holder for this preprint (which was not certified by peer review) is the author/funder, who has granted bioRxiv a license to display the preprint in perpetuity. It is made available under aCC-BY 4.0 International license.

Supplementary Material

Supplementary material includes:

Figures S1-S9

Supplementary Tables S1-S9:

Table S1: Characteristics of PLWH included in *ex vivo* experiments

Table S2: RNA-seq data: differentially expressed genes (will be uploaded to a public database)

Table S3: GSVA pathway analysis (will be uploaded to a public database)

Table S4: Individual clinical trial participant characteristics

Table S5: IPDA participant characteristics

Table S6: Characteristics of participants included in caRNA and mass cytometry assays

Table S7: Time trend analysis of IPDA measures

Table S8: Spearman correlations between changes in IPDA measures from baseline to week 2 with clinical parameters at baseline (ALN group)

Table S9: Phenotypic characterization of immune cell populations for Mass Cytometry

bioRxiv preprint doi: https://doi.org/10.1101/2023.02.07.527421; this version posted February 7, 2023. The copyright holder for this preprint (which was not certified by peer review) is the author/funder, who has granted bioRxiv a license to display the preprint in perpetuity. It is made available under aCC-BY 4.0 International license.

Supplementary Table 1. Characteristics of PLWH included in *ex vivo* experiments

N	23
Median age (years) (range)	51 (36-59)
Female (%)	3 (13.6%)
Race (%)	
African-American	7 (31.8%)
Caucasian	14 (63.6%)
Hispanic	1 (4.5%)
Median nadir CD4 count (cells/mm^3)[a] (range)	474 (195-528)
Median CD4 count (cells/mm^3) (range)	722 (605-941)
Median CD8 count (cells/mm^3)[b] (range)	622 (464-760)
Treated in acute HIV infection (%)	5 (22.7%)
Time on ART (years) (range)	5.2 (2.7-6.9)
Time suppressed (years)[c] (range)	2.5 (1.0-6.6)

Available in a) 19, b) 20, and c) 21 individuals. Due to rounding, percentages listed in the table may not sum to 100% exactly.

bioRxiv preprint doi: https://doi.org/10.1101/2023.02.07.527421; this version posted February 7, 2023. The copyright holder for this preprint (which was not certified by peer review) is the author/funder, who has granted bioRxiv a license to display the preprint in perpetuity. It is made available under aCC-BY 4.0 International license.

Supplementary Table 4. Individual clinical trial participant characteristics

Group	Pt.	Sex	Race/Ethnicity	Age	CD4 counts	CD8 counts	CD4/CD8 ratio	Years on ART
ALN	101	F	Black Non-Hispanic	48	974	669	1.46	1.46
ALN	102	F	Black Non-Hispanic	43	492	1132	0.43	4.74
ALN	103	M	White Non-Hispanic	44	679	868	0.78	9.53
ALN	104	F	White Non-Hispanic	39	687	1061	0.65	5.49
ALN	105	M	White Non-Hispanic	55	408	957	0.43	2.18
ALN	106	F	More than one race	33	153	484	0.32	0.63
ALN	107	M	White Non-Hispanic	48	429	891	0.48	5.3
ALN	108	M	White Non-Hispanic	58	1290	1935	0.67	1.44
ALN	109	F	Hispanic (Regardless of Race)	48	257	971	0.26	2.24
ALN	110	F	Black Non-Hispanic	49	232	506	0.46	4.93
ALN	111	M	White Non-Hispanic	62	1216	1687	0.72	1.9
ALN	112	F	Asian, Pacific Islander	54	219	804	0.27	0.51
ALN	113	M	White Non-Hispanic	47	559	505	1.11	0.19
ALN	114	M	Black Non-Hispanic	56	307	725	0.42	1.26
ALN	115	M	White Non-Hispanic	54	876	1169	0.75	16.8
ALN	116	M	Black Non-Hispanic	48	864	1620	0.53	2.36
ALN	117	M	White Non-Hispanic	49	464	1824	0.25	5.06
ALN	118	M	Asian, Pacific Islander	52	105	1328	0.08	1.6
ALN	119	F	Asian, Pacific Islander	52	664	708	0.94	4.31
ALN	120	M	White Non-Hispanic	47	269	1366	0.20	0.83
ALN	121	F	Hispanic (Regardless of Race)	60	906	1324	0.68	1.84
ALN	122	M	White Non-Hispanic	42	1117	549	2.03	0.93
ALN	123	M	White Non-Hispanic	63	262	759	0.35	0.85
**Median**				49	492	957	0.48	1.9

Group	Pt.	Sex	Race/Ethnicity	Age	CD4 counts	CD8 counts	CD4/CD8 ratio	Years on ART
Placebo	201	M	Black Non-Hispanic	40	653	1448	0.45	2.06
Placebo	202	M	Hispanic (Regardless of Race)	55	491	1601	0.31	0.57
Placebo	203	M	White Non-Hispanic	53	412	1510	0.27	2.01
Placebo	204	F	White Non-Hispanic	33	460	841	0.55	1.75
Placebo	205	F	Black Non-Hispanic	36	638	723	0.88	1.29
Placebo	206	M	White Non-Hispanic	42	483	372	1.30	5.07
Placebo	207	M	White Non-Hispanic	30	356	1449	0.25	0.72
Placebo	208	M	White Non-Hispanic	56	416	814	0.51	3.11
Placebo	209	M	White Non-Hispanic	57	1003	1405	0.71	4.72
Placebo	210	F	White Non-Hispanic	58	556	391	1.42	4.4
Placebo	211	F	Black Non-Hispanic	60	396	1328	0.30	0.83
Placebo	212	M	Hispanic (Regardless of Race)	45	221	488	0.45	7.31
Placebo	213	M	White Non-Hispanic	35	212	570	0.37	0.86
Placebo	214	M	White Non-Hispanic	43	493	775	0.64	9.88
Placebo	215	F	Hispanic (Regardless of Race)	45	465	581	0.80	3

bioRxiv preprint doi: https://doi.org/10.1101/2023.02.07.527421; this version posted February 7, 2023. The copyright holder for this preprint (which was not certified by peer review) is the author/funder, who has granted bioRxiv a license to display the preprint in perpetuity. It is made available under aCC-BY 4.0 International license.

Placebo	216	M	White Non-Hispanic	55	375	666	0.56	1.04
Placebo	217	F	White Non-Hispanic	68	189	192	0.98	5.13
Placebo	218	M	White Non-Hispanic	35	739	1443	0.51	2.13
Placebo	219	F	Black Non-Hispanic	41	810	718	1.13	8.16
Placebo	220	M	White Non-Hispanic	44	327	785	0.42	7.33
Placebo	221	M	White Non-Hispanic	41	1037	754	1.38	5.31
**Median**				44	465	775	0.55	3

Grey shaded samples were used for IPDA.

bioRxiv preprint doi: https://doi.org/10.1101/2023.02.07.527421; this version posted February 7, 2023. The copyright holder for this preprint (which was not certified by peer review) is the author/funder, who has granted bioRxiv a license to display the preprint in perpetuity. It is made available under aCC-BY 4.0 International license.

Supplementary Table 5. IPDA participant characteristics

	**ALN (n=9)**	**Placebo (n=7)**	**p-value[a]**
Biological sex, No. (%)			0.173
Female	1 (11.1%)	3 (42.9%)	
Male	8 (88.9%)	4 (57.1%)	
Race/Ethnicity, No. (%)			**0.033**
White non-Hispanic	5 (55.6%)	4 (57.1%)	
Black non-Hispanic	2 (22.2%)	1 (14.3%)	
Hispanic (regardless of race)	0 (0.0%)	2 (28.6%)	
Asian, Pacific Islander	2 (22.2%)	0 (0.0%)	
Age (years)[b]	49 (47 - 56)	45 (35 - 68)	0.596
CD4 count (cells/mm^3)[b]	464 (105 - 876)	375 (189 - 493)	0.204
CD8 count (cells/mm^3)[b]	1169 (505 - 1824)	581 (192 - 1328)	**0.039**
CD4/CD8 ratio[b]	0.48 (0.08 - 1.11)	0.56 (0.30 - 0.98)	0.597
Years on ART[b]	2.36 (0.19 - 16.75)	3.00 (0.83 - 9.88)	0.691

[a]P-values are calculated using Fisher's exact test (categorical variables) or Mann-Whitney U test (continuous variables). [b]Median and (range) are presented.

bioRxiv preprint doi: https://doi.org/10.1101/2023.02.07.527421; this version posted February 7, 2023. The copyright holder for this preprint (which was not certified by peer review) is the author/funder, who has granted bioRxiv a license to display the preprint in perpetuity. It is made available under aCC-BY 4.0 International license.

Supplementary Table 6. Characteristics of participants included in caRNA and mass cytometry assays

	ALN (n=15)	Placebo (n=14)	p-value[a]
Biological sex, No. (%)			
Female	8 (0.53%)	4 (0.29%)	
Male	7 (0.47%)	10 (0.71%)	
Race, No. (%)			
White non-Hispanic	8 (0.53%)	10 (0.71%)	
Black non-Hispanic	1 (0.07%)	3 (0.21%)	
Hispanic (regardless of race)	2 (0.13%)	1 (0.07%)	
Asian, Pacific Islander	1 (0.07%)	0	
Age (years)[b]	48 (43-55)	48 (35-56)	0.62
CD4 count (cells/mm^3)[b]	461 (238-902)	483 (412-638)	0.88
CD8 count (cells/mm^3)[b]	924 (703-1114)	1309 (723-1449)	0.79
Years on ART[b]	2.5 (1.4-5.2)	2.0 (1.3-4.7)	0.69

[a]P-values are calculated using Fisher's exact test (categorical variables) or Mann-Whitney U test (continuous variables). [b]Median and (range) are displayed.

bioRxiv preprint doi: https://doi.org/10.1101/2023.02.07.527421; this version posted February 7, 2023. The copyright holder for this preprint (which was not certified by peer review) is the author/funder, who has granted bioRxiv a license to display the preprint in perpetuity. It is made available under aCC-BY 4.0 International license.

Supplementary Table 7. Time trend analysis of IPDA measures

Measure	Kendall's Tau	p-Value
ALN - week 0 to 48		
Total HIV DNA (copies/10^6 PBMC)	-0.23	0.092
Total defective HIV DNA (copies/10^6 PBMC)	-0.24	0.074
3' defective HIV DNA (copies/10^6 PBMC)	**-0.30**	**0.026**
5' defective HIV DNA (copies/10^6 PBMC)	-0.20	0.144
Intact HIV DNA (copies/10^6 PBMC)	-0.05	0.735
Placebo - week 0 to 48		
Total HIV DNA (copies/10^6 PBMC)	-0.24	0.127
Total defective HIV DNA (copies/10^6 PBMC)	-0.22	0.152
3' defective HIV DNA (copies/10^6 PBMC)	-0.25	0.115
5' defective HIV DNA (copies/10^6 PBMC)	-0.20	0.199
Intact HIV DNA (copies/10^6 PBMC)	-0.11	0.482

[a]Kendall's tau is a rank-based measure of correlation. [b]p-values calculated by the Mann-Kendall test for trend. Abbreviations: IPDA, intact proviral DNA assay; PBMC, peripheral blood mononuclear cells.

bioRxiv preprint doi: https://doi.org/10.1101/2023.02.07.527421; this version posted February 7, 2023. The copyright holder for this preprint (which was not certified by peer review) is the author/funder, who has granted bioRxiv a license to display the preprint in perpetuity. It is made available under aCC-BY 4.0 International license.

Supplementary Table 8. Spearman correlations between changes in IPDA measures from baseline to week 2 with clinical parameters at baseline (ALN group)

**Clinical parameter**	r_s	**p-value**	**n**
Intact HIV DNA change[a] correlation			
CD4 count (cells/mm^3)	0.57	0.180	7
CD8 count (cells/mm^3)	-0.71	0.071	7
CD4/CD8 ratio	0.71	0.071	7
Years on ART	-0.04	0.939	7
Age	0.35	0.443	7
Defective HIV DNA change correlation			
CD4 count (cells/mm^3)	0.50	0.171	9
CD8 count (cells/mm^3)	-0.35	0.356	9
CD4/CD8 ratio	0.62	0.077	9
Years on ART	0.30	0.433	9
Age	-0.09	0.812	9
Total HIV DNA change correlation			
CD4 count (cells/mm^3)	0.50	0.171	9
CD8 count (cells/mm^3)	-0.35	0.356	9
CD4/CD8 ratio	0.62	0.077	9
Years on ART	0.30	0.433	9
Age	-0.09	0.812	9

[a]Change from 0 intact copies to 0 intact copies excluded from analysis

Abbreviations: IPDA, intact proviral DNA assay; ALN, alendronate; ART, antiretroviral therapy.

bioRxiv preprint doi: https://doi.org/10.1101/2023.02.07.527421; this version posted February 7, 2023. The copyright holder for this preprint (which was not certified by peer review) is the author/funder, who has granted bioRxiv a license to display the preprint in perpetuity. It is made available under aCC-BY 4.0 International license.

Supplementary Table S9. Phenotypic characterization of immune cell populations for Mass Cytometry**.**

**Cell subset**	**Description**
Vδ2 T cell	CD3+ CD19- CD33-, TCRvd1- TCRvd2+
Vδ1 T cell	CD3+ CD19- CD33-, TCRvd1+ TCRvd2-
CD8+ T cells (naïve)	CD3+ CD19- CD33-, TCRvd1- TCRvd2-, CD4- CD8+ CD56-, CD45RA+, CCR7+
CD8+ T cells (Effector Memory)	CD3+ CD19- CD33-, TCRvd1- TCRvd2-, CD4- CD8+ CD56-, CD45RA-, CCR7-
CD8+ T cells (Central Memory)	CD3+ CD19- CD33-, TCRvd1- TCRvd2-, CD4- CD8+ CD56-, CD45RA-, CCR7+
CD4+ T cells (naïve)	CD3+ CD19- CD33-, TCRvd1- TCRvd2-, CD4+ CD8- CD56-, CD45RA+, CCR7+
CD4+ T cells (Effector Memory)	CD3+ CD19- CD33-, TCRvd1- TCRvd2-, CD4+ CD8- CD56-, CD45RA-, CCR7-
CD4+ T cells (Central Memory)	CD3+ CD19- CD33-, TCRvd1- TCRvd2-, CD4+ CD8- CD56-, CD45RA-, CCR7+
NK cells (CD56+ CD16-)	CD3- CD19- CD33-, CD56+ CD123- HLADR-, CD16-
NK cells (CD56+ CD16+)	CD3- CD19- CD33-, CD56+ CD123- HLADR-, CD16+
Dendritic cells	CD3- CD19- CD33+, CD14-, CD16-
Plasmacytoid dendritic cells	CD3- CD19- CD33-, CD11c- CD123+ HLADR+
Monocytes	CD3- CD19- CD33+, CD14+, CD16-
B cells	CD3- CD19+ CD33-

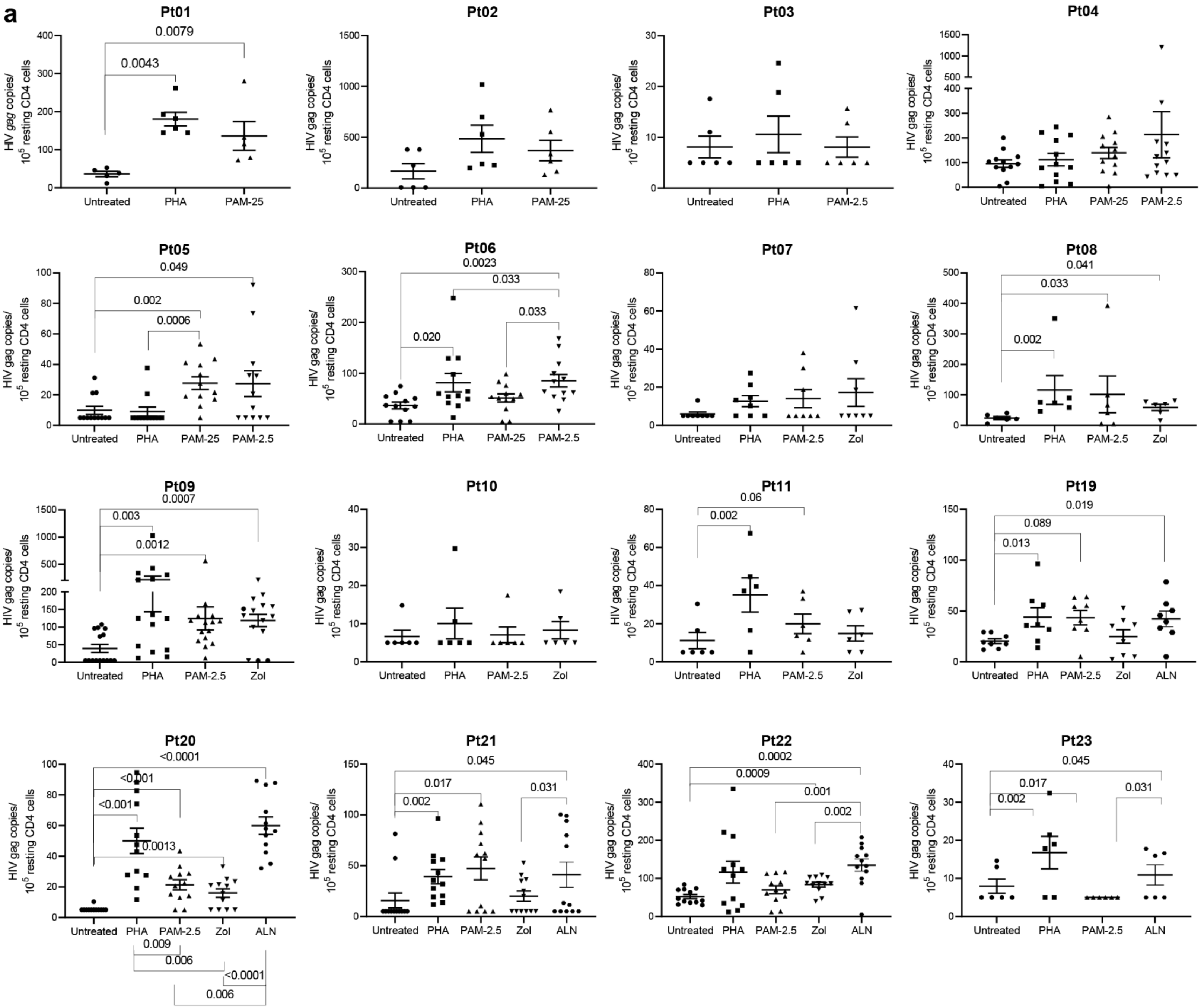
a
Pt01
0.0079
0.0043
HIV gag copies/
10^5 resting CD4 cells
Untreated
PHA
PAM-25
Pt02
Untreated
PHA
PAM-25
Pt03
Untreated
PHA
PAM-2.5
Pt04
Untreated
PHA
PAM-25
PAM-2.5
Pt05
0.049
0.002
0.0006
Untreated
PHA
PAM-25
PAM-2.5
Pt06
0.0023
0.033
0.033
0.020
Untreated
PHA
PAM-25
PAM-2.5
Pt07
Untreated
PHA
PAM-2.5
Zol
Pt08
0.041
0.033
0.002
Untreated
PHA
PAM-2.5
Zol
Pt09
0.0007
0.003
0.0012
Untreated
PHA
PAM-2.5
Zol
Pt10
Untreated
PHA
PAM-2.5
Zol
Pt11
0.06
0.002
Untreated
PHA
PAM-2.5
Zol
Pt19
0.019
0.089
0.013
Untreated
PHA
PAM-2.5
Zol
ALN
Pt20
<0.0001
<0.001
<0.001
0.0013
Untreated
PHA
PAM-2.5
Zol
ALN
0.009
0.006
<0.0001
0.006
Pt21
0.045
0.017
0.031
0.002
Untreated
PHA
PAM-2.5
Zol
ALN
Pt22
0.0002
0.0009
0.001
0.002
Untreated
PHA
PAM-2.5
Zol
ALN
Pt23
0.045
0.017
0.031
0.002
Untreated
PHA
PAM-2.5
ALN

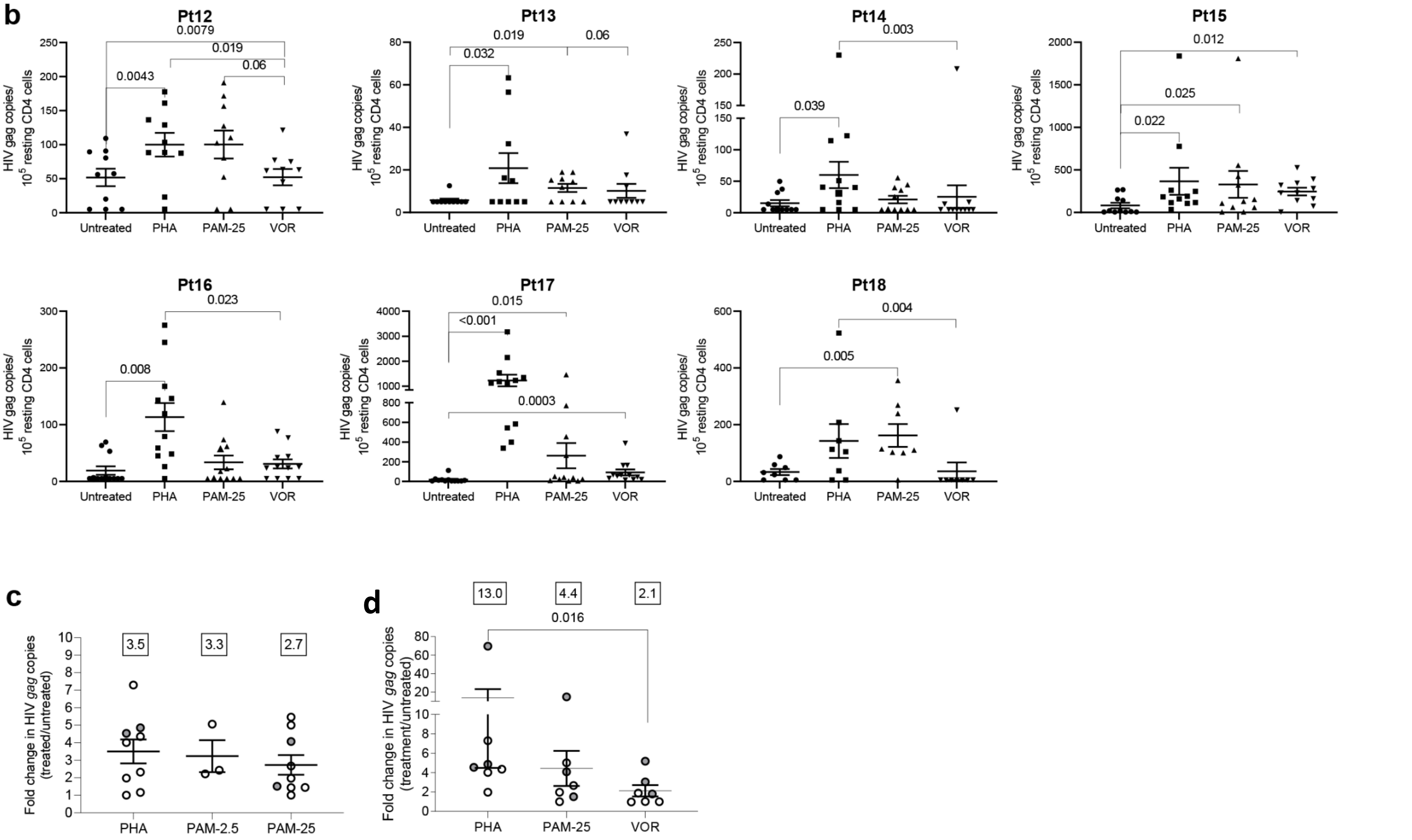

Figure S1: HIV-1 caRNA levels in 23 PLWH on suppressive ART. Individual plots show HIV-1 *gag* caRNA copies/mL in the different conditions tested in each experiment depending on cell availability. **A)** Comparison of PAM, Zol and ALN with PHA. **B)** In Pt12 through Pt18, the capacity of N-BPs to reactivate latent HIV was compared to 500nM VOR. Each graph represents one participant, and each symbol represents one biological replicate of $1x10^6$ isolated rCD4 T cells. Comparison of HIV gag copies induction between **C)** PAM at 2.5µg/mL and 25µg/mL) and **D)** PAM at 25µg/mL and VOR. Pt, participant. P-values were calculated by a Wilcoxon signed-rank test, and only p-values < 0.05 are displayed.

bioRxiv preprint doi: https://doi.org/10.1101/2023.02.07.527421; this version posted February 7, 2023. The copyright holder for this preprint (which was not certified by peer review) is the author/funder, who has granted bioRxiv a license to display the preprint in perpetuity. It is made available under aCC-BY 4.0 International license.

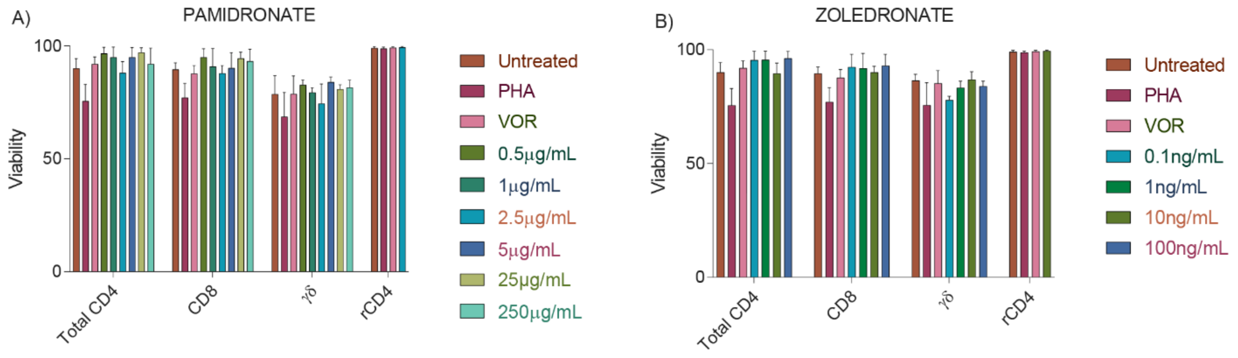

Figure S2: ***Ex vivo*** **viability upon treatment with PAM and Zol.** PBMCs from PLWH on suppressed ART were exposed to 2 μg/mL PHA+100 U/mL IL-2 or different concentrations of **A)** PAM or **B)** Zol as specified, and viability (7-AAD) measured by flow cytometry in total CD4 T cells, CD8 T cells, γδ T cells, and resting CD4 (rCD4) T cells. The means of repeated experiments in three to six individuals are presented.

bioRxiv preprint doi: https://doi.org/10.1101/2023.02.07.527421; this version posted February 7, 2023. The copyright holder for this preprint (which was not certified by peer review) is the author/funder, who has granted bioRxiv a license to display the preprint in perpetuity. It is made available under aCC-BY 4.0 International license.

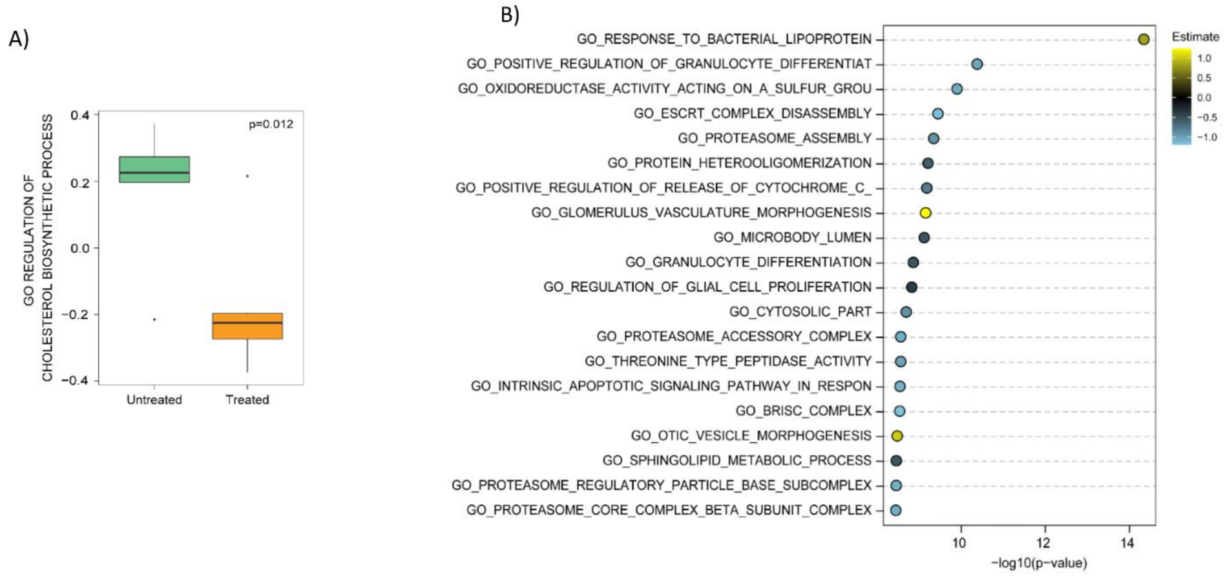

Figure S3: A) Boxplot of enrichment score derived from gene set variation analysis (GSVA) of GO annotation of Cholesterol Biosynthesis Regulation comparing untreated and PAM-treated individuals (p=0.012). **B)** Top 20 GO annotations score derived from gene set variation analysis (GSVA) of GO annotation between the untreated and PAM-treated samples. Estimates (coefficients of the predictor treatment) are sorted (on the x-axis) by -log10(p-value) in decreasing direction and color-coded based on the value of the estimate. Yellow: positive estimates; Blue: negative estimates.

bioRxiv preprint doi: https://doi.org/10.1101/2023.02.07.527421; this version posted February 7, 2023. The copyright holder for this preprint (which was not certified by peer review) is the author/funder, who has granted bioRxiv a license to display the preprint in perpetuity. It is made available under aCC-BY 4.0 International license.

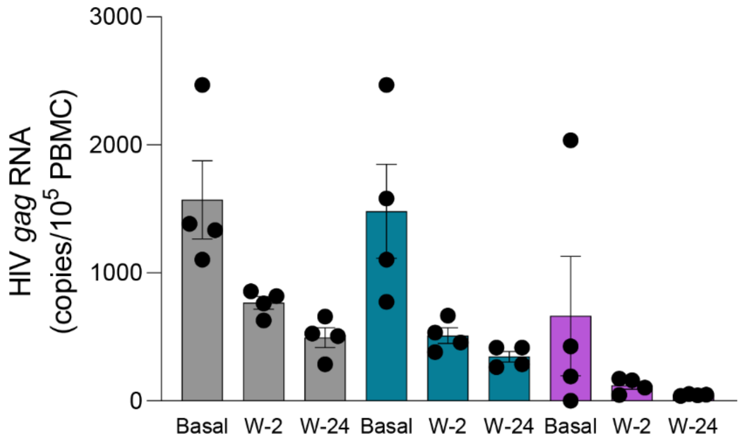

Figure S4: HIV DNA levels in participant 107 at baseline and after ALN treatment. Mean± SEM is represented. Hypermutated 3'defective (grey), 5' defective (blue) and intact provirus (purple) are presented.

bioRxiv preprint doi: https://doi.org/10.1101/2023.02.07.527421; this version posted February 7, 2023. The copyright holder for this preprint (which was not certified by peer review) is the author/funder, who has granted bioRxiv a license to display the preprint in perpetuity. It is made available under aCC-BY 4.0 International license.

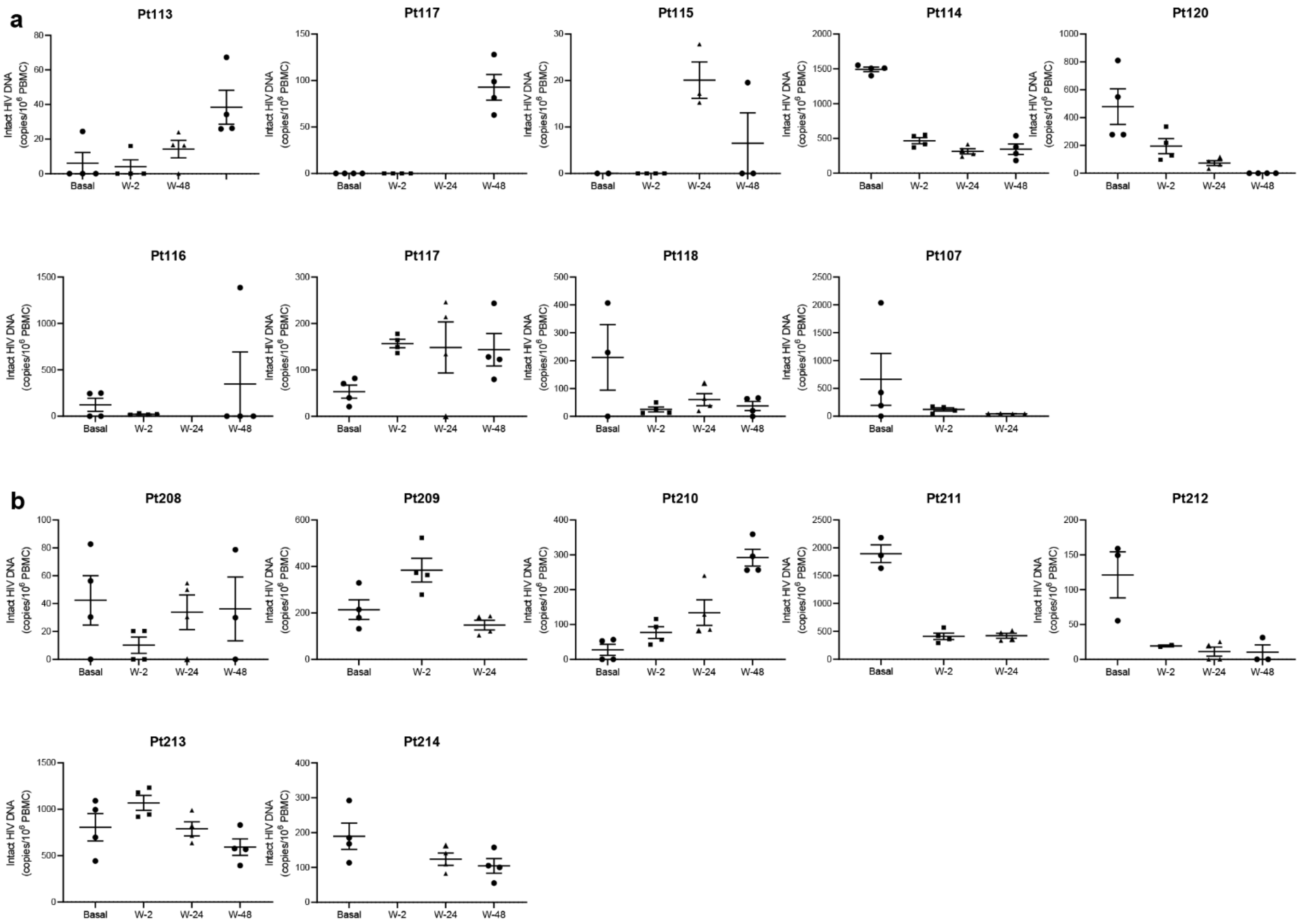

bioRxiv preprint doi: https://doi.org/10.1101/2023.02.07.527421; this version posted February 7, 2023. The copyright holder for this preprint (which was not certified by peer review) is the author/funder, who has granted bioRxiv a license to display the preprint in perpetuity. It is made available under aCC-BY 4.0 International license.

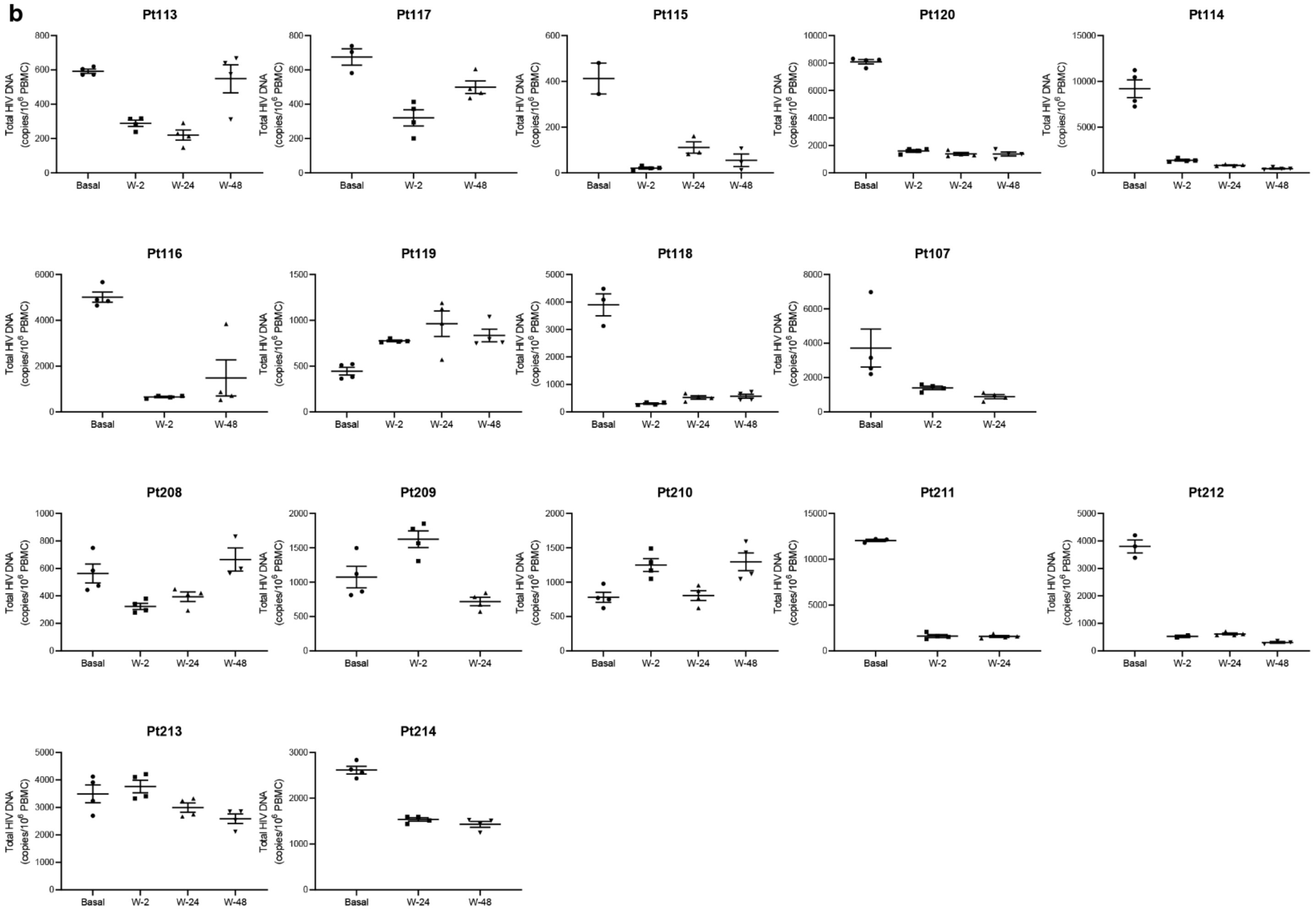

Figure S5: Individual IPDA data showing intact provirus in **A)** ALN and **B)** placebo groups, and total HIV-DNA in **C)** ALN and **D)** placebo groups.

bioRxiv preprint doi: https://doi.org/10.1101/2023.02.07.527421; this version posted February 7, 2023. The copyright holder for this preprint (which was not certified by peer review) is the author/funder, who has granted bioRxiv a license to display the preprint in perpetuity. It is made available under aCC-BY 4.0 International license.

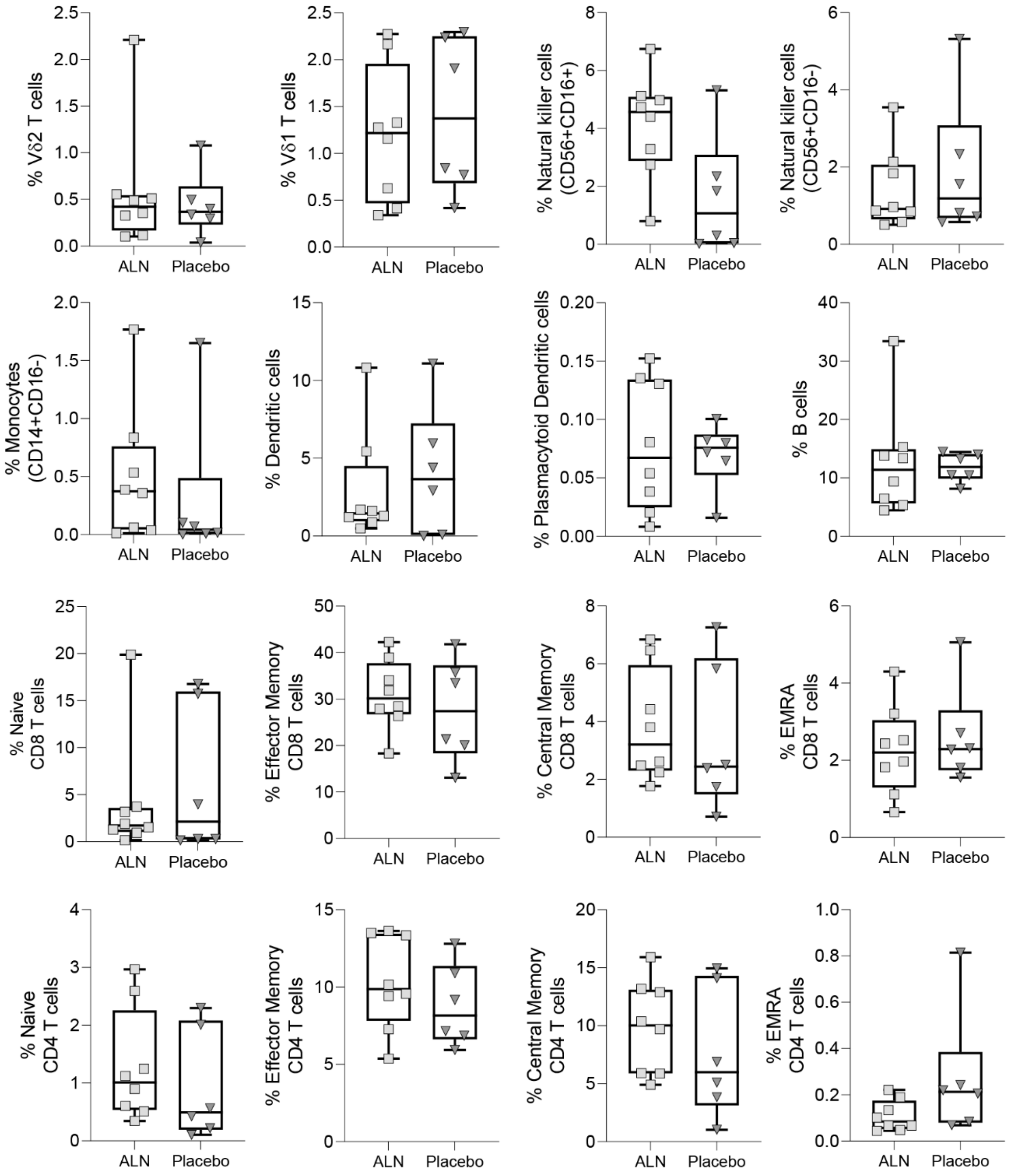

Figure S6: Comparison of basal cell frequencies between individuals who took ALN and placebo. Mass cytometry performed on eight individuals from the ALN group and six from the placebo group. Boxplots display first quartile, median, and third quartile with whiskers range from the minimum to maximum values. Mann-Whitney U test p-values>0.05 for all comparisons between ALN and placebo

bioRxiv preprint doi: https://doi.org/10.1101/2023.02.07.527421; this version posted February 7, 2023. The copyright holder for this preprint (which was not certified by peer review) is the author/funder, who has granted bioRxiv a license to display the preprint in perpetuity. It is made available under aCC-BY 4.0 International license.

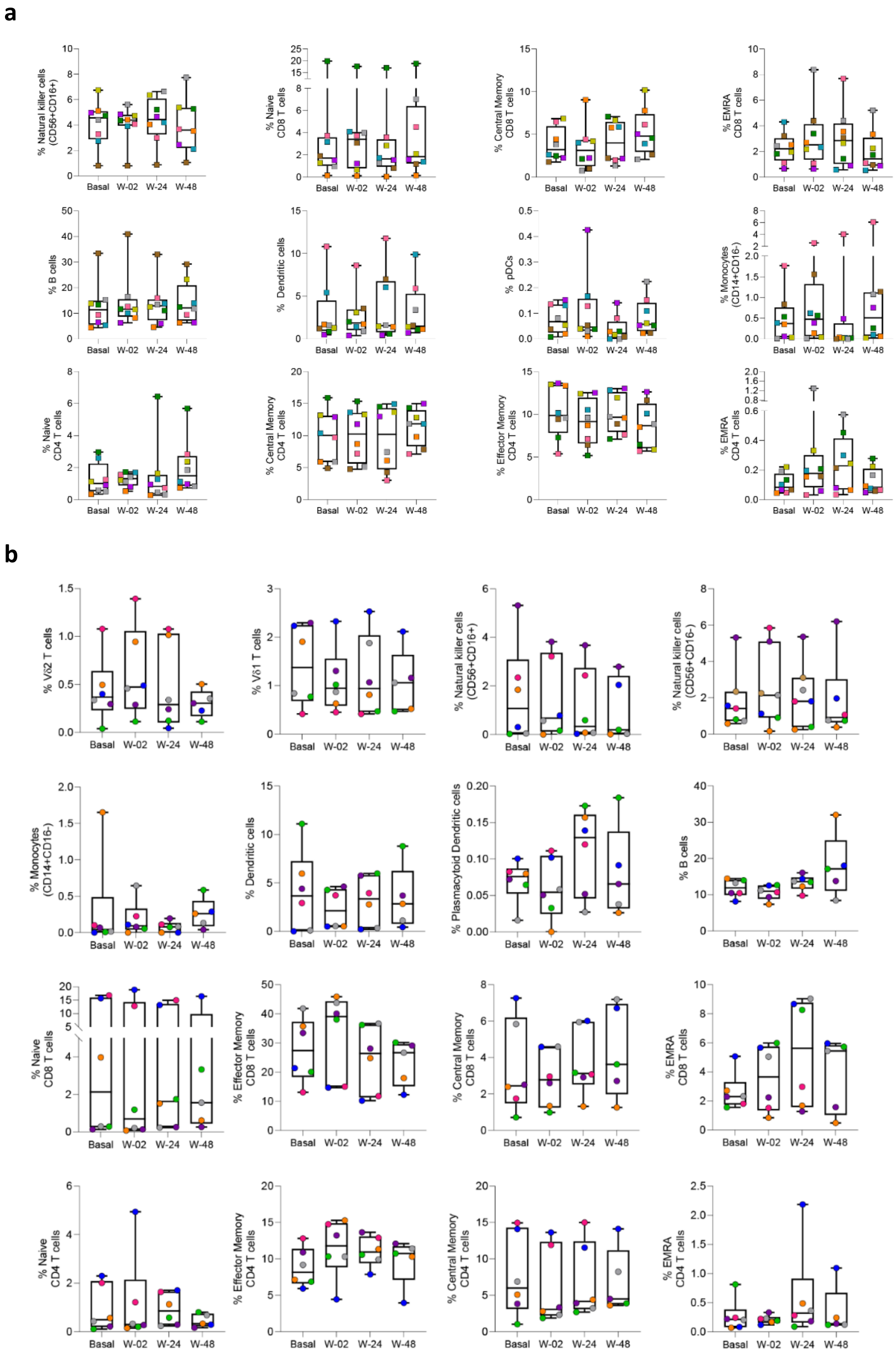

Figure S7: Longitudinal frequencies of circulating cell populations in participants from the A) ALN and B) placebo groups. Wilcoxon signed-rank p-values > 0.05 for all comparisons and adjusted for multiple comparisons using Holm-Bonferroni.

bioRxiv preprint doi: https://doi.org/10.1101/2023.02.07.527421; this version posted February 7, 2023. The copyright holder for this preprint (which was not certified by peer review) is the author/funder, who has granted bioRxiv a license to display the preprint in perpetuity. It is made available under aCC-BY 4.0 International license.

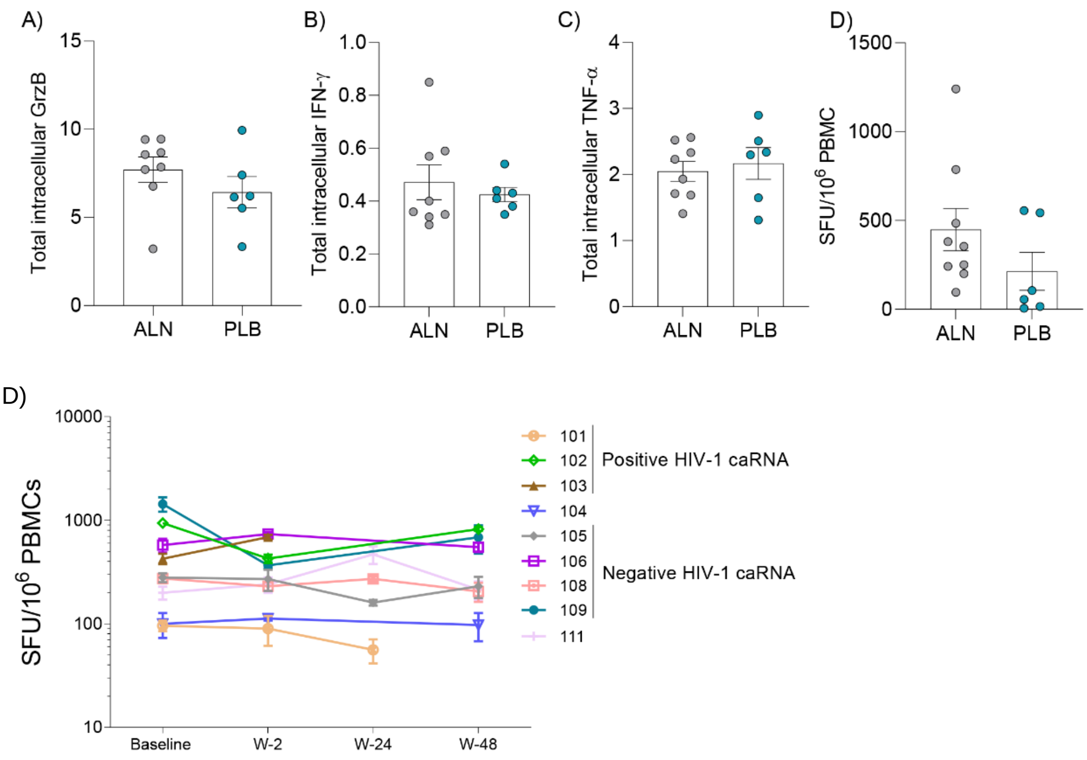

Figure S8: Intracellular functional markers in participants from the ALN and placebo groups. Baseline levels of intracellular production of **A)** granzyme (Grz) B, **B)** IFN-γ, **C)** TNF-α, and **D)** Baseline spot forming units (SFU)/10^6 PBMCs were comparable between participants who took ALN and PLB (Mann-Whitney U test). **D)** SFU/10^6 PBMC over time in participants treated with ALN, with legend denoting those with negative versus positive trend in HIV-1 caRNA levels.

bioRxiv preprint doi: https://doi.org/10.1101/2023.02.07.527421; this version posted February 7, 2023. The copyright holder for this preprint (which was not certified by peer review) is the author/funder, who has granted bioRxiv a license to display the preprint in perpetuity. It is made available under aCC-BY 4.0 International license.

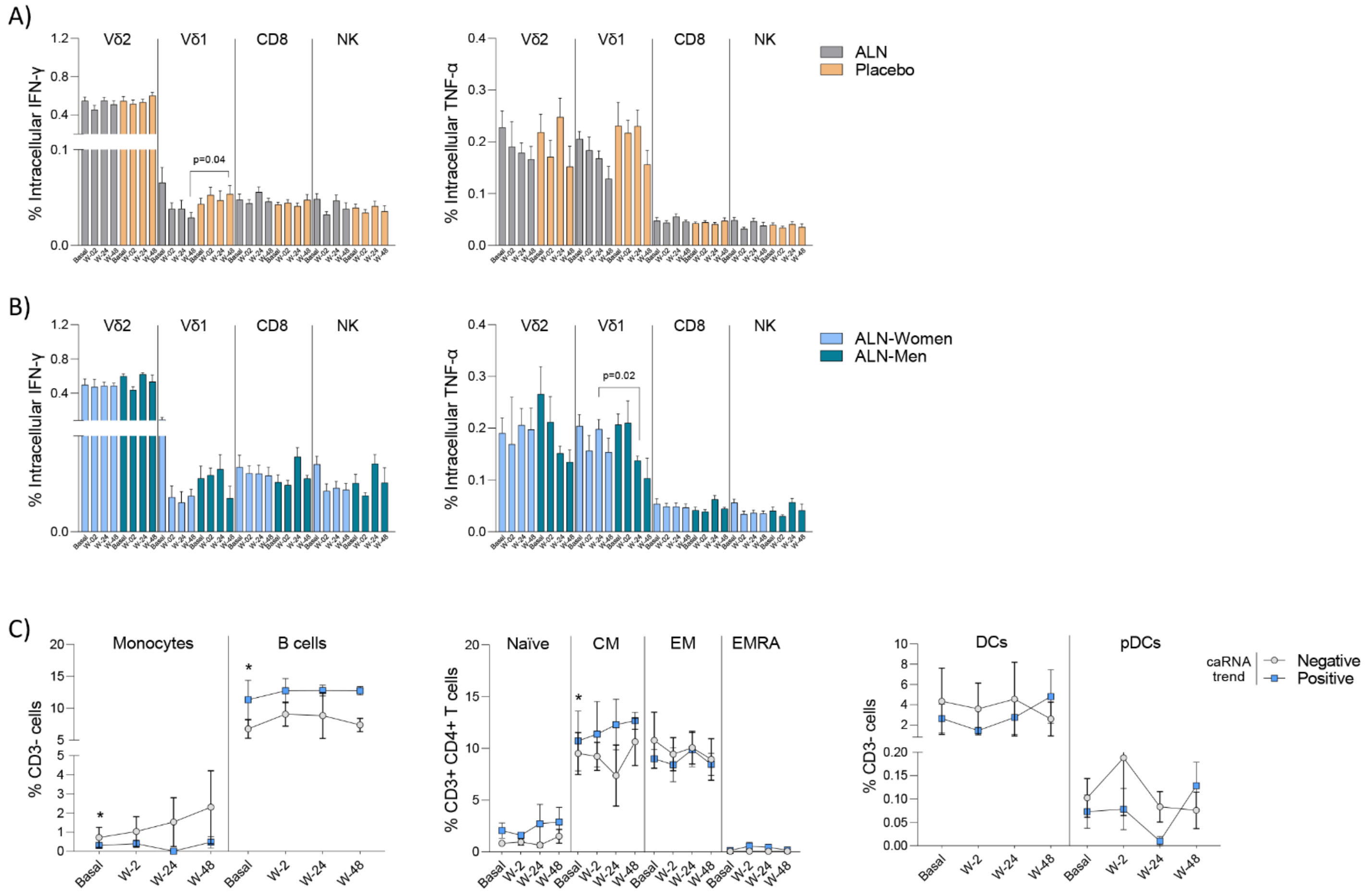

Figure S9: Comparison of IFN-γ and TNF-α production from different effector cell populations (N=8) in A) ALN or placebo groups, B) women and men treated with ALN. C) Comparison of frequencies of circulating cell populations according to the HIV caRNA slope (N=3 negative trend, grey, and N=3 positive trend, blue). Mean ±SEM is presented. Mann-Whitney U test.
